# The Canadian VirusSeq Data Portal and Duotang: open resources for SARS-CoV-2 viral sequences and genomic epidemiology

**DOI:** 10.1099/mgen.0.001293

**Published:** 2024-10-14

**Authors:** Erin E. Gill, Baofeng Jia, Carmen Lia Murall, Raphaël Poujol, Muhammad Zohaib Anwar, Nithu Sara John, Justin Richardsson, Ashley Hobb, Abayomi S. Olabode, Alexandru Lepsa, Ana T. Duggan, Andrea D. Tyler, Arnaud N'Guessan, Atul Kachru, Brandon Chan, Catherine Yoshida, Christina K. Yung, David Bujold, Dusan Andric, Edmund Su, Emma J. Griffiths, Gary Van Domselaar, Gordon W. Jolly, Heather K. E. Ward, Henrich Feher, Jared Baker, Jared T. Simpson, Jaser Uddin, Jiannis Ragoussis, Jon Eubank, Jörg H. Fritz, José Héctor Gálvez, Karen Fang, Kim Cullion, Leonardo Rivera, Linda Xiang, Matthew A. Croxen, Mitchell Shiell, Natalie Prystajecky, Pierre-Olivier Quirion, Rosita Bajari, Samantha Rich, Samira Mubareka, Sandrine Moreira, Scott Cain, Steven G. Sutcliffe, Susanne A. Kraemer, Yelizar Alturmessov, Yann Joly, CPHLN Consortium**, CanCOGeN Consortium**, VirusSeq Data Portal Academic and Health Network**, Marc Fiume, Terrance P. Snutch, Cindy Bell, Catalina Lopez-Correa, Julie G. Hussin, Jeffrey B. Joy, Caroline Colijn, Paul M. K. Gordon, William W. L. Hsiao, Art F. Y. Poon, Natalie C. Knox, Mélanie Courtot, Lincoln Stein, Sarah P. Otto, Guillaume Bourque, B. Jesse Shapiro, Fiona S. L. Brinkman, CPHLN consortium

**Affiliations:** 1 Department of Molecular Biology and Biochemistry, Simon Fraser University, Burnaby, BC, Canada; 2 Department of Microbiology and Immunology, McGill University, Montreal, QC, Canada; 3 National Microbiology Laboratory, Public Health Agency of Canada, Winnipeg, MB, Canada; 4 Research Centre, Montréal Heart Institute, Montréal, QC, Canada; 5 Centre for Infectious Disease Genomics and One Health, Faculty of Health Sciences, Simon Fraser University, Burnaby, BC, Canada; 6 Ontario Institute for Cancer Research, Toronto, ON, Canada; 7 DNAstack, Toronto, ON, Canada; 8 Department of Pathology and Laboratory Medicine, Western University, London, ON, Canada; 9 Département de Biochimie et Médecine Moléculaire, Université de Montréal, Montreal, QC, Canada; 10 McGill Genome Centre, McGill University, Montréal, QC, Canada; 11 Indoc Systems, Toronto, ON, Canada; 12 Department of Human Genetics, McGill University, Montréal, QC, Canada; 13 Canadian Centre for Computational Genomics, Montréal, QC, Canada; 14 Department of Microbiology and Immunology, McGill Research Center on Complex Traits (MRCCT), Dahdaleh Institute of Genomic Medicine (DIGM), McGill University, Montréal, QC, Canada; 15 Alberta Precision Laboratories, Public Health Laboratory, Edmonton, AB, Canada; 16 Department of Laboratory Medicine and Pathology, University of Alberta, Edmonton, AB, Canada; 17 Li Ka Shing Institute of Virology, University of Alberta, Edmonton, AB, Canada; 18 Women and Children’s Health Research Institute, University of Alberta, Edmonton, AB, Canada; 19 British Columbia Centre for Disease Control Public Health Laboratory, Vancouver, BC, Canada; 20 Department of Pathology and Laboratory Medicine, Faculty of Medicine, University of British Columbia, Vancouver, BC, Canada; 21 Sunnybrook Research Institute, Department of Laboratory Medicine and Pathobiology, University of Toronto, Toronto, ON, Canada; 22 Université de Montréal, Montréal, QC, Canada; 23 Department of Microbiology and Immunology, McGill University, Montréal, QC, Canada; 24 Aquatic Contaminants Research Division, ECCC, Montréal, QC, Canada; 25 Centre of Genomics and Policy, McGill University, Montréal, QC, Canada; 26 Michael Smith Laboratories and Djavad Mowafaghian Centre for Brain Health, University of British Columbia, Vancouver, BC, Canada; 27 Genome Canada, 150 Metcalfe Street, Suite 2100, Ottawa, ON, Canada; 28 Research Centre, Montréal Heart Institute, Montréal, QC, Canada; 29 Mila-Québec AI institute, Montréal, QC, Canada; 30 Molecular Epidemiology and Evolutionary Genetics, BC Centre for Excellence in HIV/AIDS, Vancouver, BC, Canada; 31 Infectious Diseases, Department of Medicine, University of British Columbia, Vancouver, BC, Canada; 32 Bioinformatics Programme, University of British Columbia, Vancouver, BC, Canada; 33 Department of Mathematics, Simon Fraser University, Burnaby, BC, Canada; 34 Centre for Health Genomics and Informatics, University of Calgary, Calgary, AB, Canada; 35 Department of Medical BioPhysics, University of Toronto, ON, Canada; 36 Department of Zoology and Biodiversity Research Centre, University of British Columbia, Vancouver, BC, Canada

**Keywords:** data sharing, evolutionary biology, mutational analysis, open access, viral genomics

## Abstract

The COVID-19 pandemic led to a large global effort to sequence SARS-CoV-2 genomes from patient samples to track viral evolution and inform the public health response. Millions of SARS-CoV-2 genome sequences have been deposited in global public repositories. The Canadian COVID-19 Genomics Network (CanCOGeN – VirusSeq), a consortium tasked with coordinating expanded sequencing of SARS-CoV-2 genomes across Canada early in the pandemic, created the Canadian VirusSeq Data Portal, with associated data pipelines and procedures, to support these efforts. The goal of VirusSeq was to allow open access to Canadian SARS-CoV-2 genomic sequences and enhanced, standardized contextual data that were unavailable in other repositories and that meet FAIR standards (Findable, Accessible, Interoperable and Reusable). In addition, the portal data submission pipeline contains data quality checking procedures and appropriate acknowledgement of data generators that encourages collaboration. From inception to execution, the portal was developed with a conscientious focus on strong data governance principles and practices. Extensive efforts ensured a commitment to Canadian privacy laws, data security standards, and organizational processes. This portal has been coupled with other resources, such as Viral AI, and was further leveraged by the Coronavirus Variants Rapid Response Network (CoVaRR-Net) to produce a suite of continually updated analytical tools and notebooks. Here we highlight this portal (https://virusseq-dataportal.ca/), including its contextual data not available elsewhere, and the Duotang (https://covarr-net.github.io/duotang/duotang.html), a web platform that presents key genomic epidemiology and modelling analyses on circulating and emerging SARS-CoV-2 variants in Canada. Duotang presents dynamic changes in variant composition of SARS-CoV-2 in Canada and by province, estimates variant growth, and displays complementary interactive visualizations, with a text overview of the current situation. The VirusSeq Data Portal and Duotang resources, alongside additional analyses and resources computed from the portal (COVID-MVP, CoVizu), are all open source and freely available. Together, they provide an updated picture of SARS-CoV-2 evolution to spur scientific discussions, inform public discourse, and support communication with and within public health authorities. They also serve as a framework for other jurisdictions interested in open, collaborative sequence data sharing and analyses.

**Significance as a BioResource to the community**

Here, we describe two new open-source, open-access resources for the scientific community. The first, the VirusSeq Data Portal, is a web portal containing SARS-CoV-2 genomic sequences derived from clinical samples collected from across Canada from the start of the pandemic to the present. Genomic data are accompanied by contextual data such as patient age, gender and sample collection date, plus additional contextual data useful for analyses that are not available elsewhere (like ‘purpose of sequencing’, which aids analysis of variant growth, by differentiating cases sequenced due to baseline surveillance, verses outbreaks, border cases, etc.). The second resource, Duotang, is an interactive webpage displaying genomic and epidemiological analyses of the sequences from the data portal. Duotang is updated on a weekly basis and presents an overview of the current COVID-19 situation in Canada. Duotang presents a combination of analyses that are (to our knowledge) not otherwise available publicly. The data portal is unique in that it provides sequence data that have undergone rigorous quality checks, and the accompanying contextual data are harmonized across Canada’s federated healthcare system, and more substantial than those available from other online databases. Beyond Canada, these tools are readily transferable to other jurisdictions interested in genomic, phylogenetic or molecular epidemiological analyses of SARS-CoV-2.

## Data Summary

VirusSeq Data Portal URL: https://virusseq-dataportal.ca/

VirusSeq Data Portal GitHub: https://github.com/virusseq/

Duotang GitHub: https://github.com/CoVaRR-NET/duotang

Duotang URL: https://covarr-net.github.io/duotang/duotang.html

DataHarmonizer GitHub: https://github.com/cidgoh/DataHarmonizer.

## Introduction

As the COVID-19 pandemic began to unfold, different countries and jurisdictions developed their own SARS-CoV-2 whole-genome sequencing capacity, quality standards, analysis pipelines, and methods to release sequence data and accompanying contextual data for public use.

In Canada, these activities were spearheaded by federal funding to Genome Canada, a not-for-profit organization. Genome Canada supported the creation of the Canadian COVID-19 Genomics Network (CanCOGeN), a Canadian consortium for sequencing SARS-CoV-2 (VirusSeq) and its human host (HostSeq) in April of 2020. This pan-Canadian network was composed of academics, national, provincial, and territorial public health labs, hospitals, research institutes, and industry. The VirusSeq component of the consortium aimed to track viral spread and evolution through the sequencing and analysis of viral genomes collected and sequenced in Canada. It relied on the participation of the Canadian public to obtain the samples and data necessary for its work. The initial goal was to sequence up to 150 000 viral genomes within 2 years (beginning from roughly 10 000 in August 2020), which was readily surpassed (150 000 sequences were completed in November 2021). As of March 2024, the network had sequenced and shared over 550 000 SARS-CoV-2 genomes. These data are increasingly used for integrated analysis of genome evolution across Canada’s jurisdictions, for example, as presented in the Duotang to identify emerging variants, estimate their growth advantage, and relate genomic trends to those observed in publicly available case statistics.

### Sequencing and data sharing

The Canadian public health system is decentralized and federated, where each province and territory has its own unique healthcare system and is a participant in the Canadian Public Health Laboratory Network (CPHLN). As part of the CanCOGeN consortium, the members of the CPHLN, as well as the Public Health Agency of Canada’s National Microbiology Laboratory (PHAC-NML) and regional health units and hospitals, strengthened their sequencing capacity and expertise. In parallel, others in the consortium worked on developing data sharing agreements and engaging the community in the adoption of effective, uniform and secure data sharing practices. For example, at the outset of the pandemic, each province and territory (and sometimes regional health authorities within provinces) used its own case report form to collect contextual data with its viral samples (e.g. patient age bracket, gender, etc.) [1]. Once samples have been sequenced by regional public health laboratories (CPHLN branches), their academic and other partners or PHAC-NML, viral genomes and accompanying data were then shared with PHAC-NML and publicly accessible databases.

The diverse formatting and differences in the types of data collected have complicated cross-jurisdictional sequence comparisons. Analyses were also hampered by the speed with which sequence data were shared to public repositories such as GISAID [2,4], which varied greatly from one region to the next and earlier in the pandemic averaged nearly 3 months across Canada (through May 2021 [5]). Support from PHAC-NML through provincial partners, including deployed Genomics Liaison Technical Officers, coordinated reagent procurement, and innovation funding, enabled accelerated sequencing and data movement to achieve and maintain a median time from collection to submission of <21 days since December 2021. While GISAID has been a powerful resource for facilitating global data integration, it is not fully open and has restrictive user access and terms of use. In the Canadian context, this means that downloading sequences from GISAID, from adjacent provinces or territories, analysing, and publicly presenting them side by side would have violated the terms of the organization, which require a lengthy process of pre-approval by the different data providers for use of unpublished data. Given some controversy that has surrounded GISAID, involving its management, transparency, and access concerns [6,7], sustaining an alternative platform for sequence data sharing has become a priority.

The VirusSeq Data Portal was launched in April 2021, when roughly 80 000 sequences had been generated. The portal is an open-access, open-source web-based resource that contains SARS-CoV-2 genome sequence data from across Canada that have been collected from the start of the pandemic to the present. The sequence data are available for download to anybody without the necessity of creating an account. Critically, the sequence data are linked with a set of contextual data, such as region of origin, date of collection, reason for sequencing, patient age bracket and gender. These contextual data are standardized so that they appear in the same format for each record, and sequence data undergo rigorous quality checks.

The VirusSeq Data Portal serves an integral purpose in that it allows data from different regions to be analysed jointly in real time. In certain cases, it also provides contextual data that are absent from other large public databases, permitting analyses not possible elsewhere. For example, a more robust evaluation of variant growth can feature in ‘purpose of sequencing’ (whether the case was due to baseline surveillance, targeted surveillance, surveillance of international border crossing, cluster/outbreak investigation, etc.). The use of a DataHarmonizer tool [8] and uploader for FASTA files facilitates more standardized data curation and aids the deposition process for data producers, so contextual data fields collected using different forms can be integrated into a single searchable database. The DataHarmonizer also simplifies sequence submission to other public databases should the data provider choose to do so.

This portal, with its focus on enriched contextual data and genomic epidemiology analyses, complements other resources developed, such as the European COVID-19 Data Portal developed by EMBL-EBI [9], which offers multiple sequence analysis workflows and enhanced variant browsing, and the California SARS-CoV-2 Whole Genome Sequencing Initiative workspace called COVIDNet [10], which also centralizes workflows, but has a focus on training sessions for public health. Data in the VirusSeq Data Portal are recorded using Public Health Alliance for Genomic Epidemiology (PHA4GE) data standards/specifications [11], to support interoperability with other international resources. While other international resources have fewer metadata, data are still uploaded to international resources to further support global efforts.

### A portal to variant discovery

With the advent of SARS-CoV-2 variants of concern (VOCs), Canada launched a funding call for a separate academic entity to tackle the emerging threat posed by new variants: the Coronavirus Variants Rapid Response Network (CoVaRR-Net). The Network was formed in early 2021 with a mandate to “coordinate, facilitate and accelerate rapid research throughout Canada that rapidly answer[s] critical […] questions regarding variants, such as their increased transmissibility”.

One CoVaRR-Net pillar, the Computational Analysis, Modelling and Evolutionary Outcomes (CAMEO) initiative, was set up to focus on computational and mathematical approaches to variant surveillance. CAMEO relies on the publicly available sequencing and contextual data in the data portal to track the emergence, introduction, and spread of VOCs within Canada. Modelling tools were developed to estimate how quickly variants might propagate in the near future and which variants were likely to overtake other lineages. These tools continue to be applied on both a regional and national basis, with reference to the global context. Potential variants of Canadian origin are flagged for follow-up, and mutations that could confer traits such as resistance to antivirals or stronger binding to the human ACE-2 receptor are tracked.

During the fourth wave (autumn 2021), CAMEO teamed up with the PHAC-NML and the Public Health laboratories under CanCOGeN to jointly track the two major Delta lineages growing in Canada: AY.25 and AY.27 [12]. The compilation of methods used for this analysis led to the idea of building a pipeline to run in a more automated fashion to improve the speed of tracking and to share the methods for use by any jurisdiction. An R Markdown notebook was chosen to accommodate both modelling and visualization of bioinformatic results with web-viewing, interactivity, and explanatory text. This notebook was named ‘Duotang’, in reference to Canadian slang for ‘workbook’ used in schools (a generalized trademark; https://en.wikipedia.org/wiki/Duo-Tang) and launched in early 2022. The accompanying GitHub repository of the network (https://github.com/CoVaRR-NET) houses code for running analyses and data visualizations, as well as recipes for running bioinformatics pipelines, such as building SARS-CoV-2 phylogenetic trees using various methods or tracking the accumulation of mutations over time. An option to password protect versions of the Duotang notebook is available, in cases where there are privacy concerns or non-public data are being analysed. The underlying code is readily generalizable to other diseases and can also be used for training purposes for students, public health staff, or new research assistants that joined the network. The tools were used to aid risk assessment for different variants, capitalizing on the federated data to identify variants of interest that, in the Canadian vaccination/immunity context, were undergoing selection in multiple provinces and so unlikely to be growing due to a founder effect or superspreader event [13].

## Methods

**Fig. 1.**
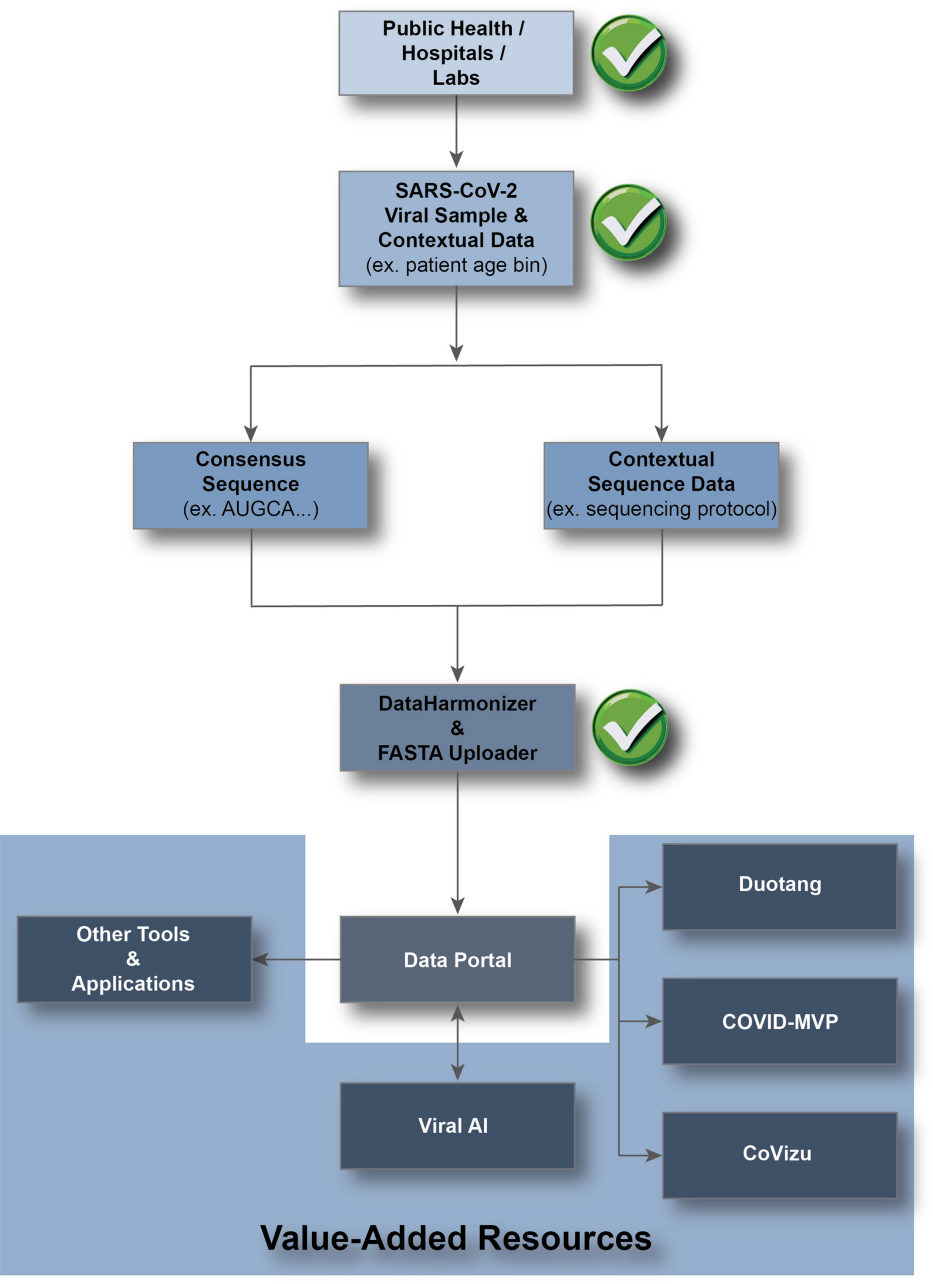
Data flow overview. SARS-CoV-2 viral samples and contextual data are collected by Public Health, hospitals and labs. Some samples are selected for sequencing based on national and regional priorities. After sequencing, regional Public Health authorities select samples that will be shared publicly. Sequence and contextual data from these samples are uploaded to the data portal using the DataHarmonizer and FASTA uploader. From the portal, the public can view or download contextual and sequence data from across the country. Data can also be accessed for value-added resources, such as Duotang, ViralAI, COVID-MVP, and CoVizu, via the API. Green check marks on the figure indicate points in the data flow where human quality assurance/quality control (QA/QC) and/or ethical oversight take place.

We briefly summarize the tools used to create the VirusSeq Data Portal, the data processing steps, and the analyses in Duotang (see https://github.com/CoVaRR-NET/duotang for Duotang code). Fig. 1 presents an overview of the data flow from sample collection to the data portal then Duotang.

### Development of the VirusSeq Data Portal

The Ontario Institute for Cancer Research’s Genome Informatics team spearheaded the development and the flexible and scalable deployment of the VirusSeq Data Portal using the Overture [14] collection of reusable software microservices, including Ego, Song, Score, Maestro, and Arranger, and Keycloak [15], a third-party OAuth service. In short, Score manages authorized file transfers to and from an S3 object storage in collaboration with Song, which validates submitted file metadata against an established data model and serves as the single source of truth (SSOT) for the data in the system. After file upload and metadata validation, with the assistance of the event-driven messaging system Kafka, Maestro indexes the information into an Elasticsearch cluster. Finally, Arranger, our GraphQL search application programming interface (API) and portal user interface (UI) generator, consumes the indexed data, making it available to the users through a customized Overture document management system–user interface. Together, Ego and Keycloak were used to facilitate the authorization and authentication of users and applications using JSON Web Tokens. In addition to the core components, specialized services (https://github.com/virusseq/) were crafted to cater to the unique requirements of the VirusSeq portal. (1) The Pedigree service was created to fetch and aggregate lineage metadata from Viral AI – a federated network for genomic variant surveillance developed by Canadian biotech company DNAStack [16] (See ‘Viral AI lineage assignment’), allowing users to search by lineage. (2) Muse was introduced to verify that each viral genome FASTA file was paired with a relevant metadata record, increasing the data quality of the resource. (3) Singularity was developed to manage ‘data release’ bundling and file downloads, enabling users to access large file numbers efficiently on release. The database containing the sequence data and the data fields that are available with each sequence have been carefully designed to prevent host reidentification. For example, geographical location of sample collection is only available at the provincial/territorial level (these regions contain hundreds of thousands of residents). This allows finer-grain reporting of fields such as ‘sample collection date’.

### Viral AI lineage assignment

**Fig. 2.**
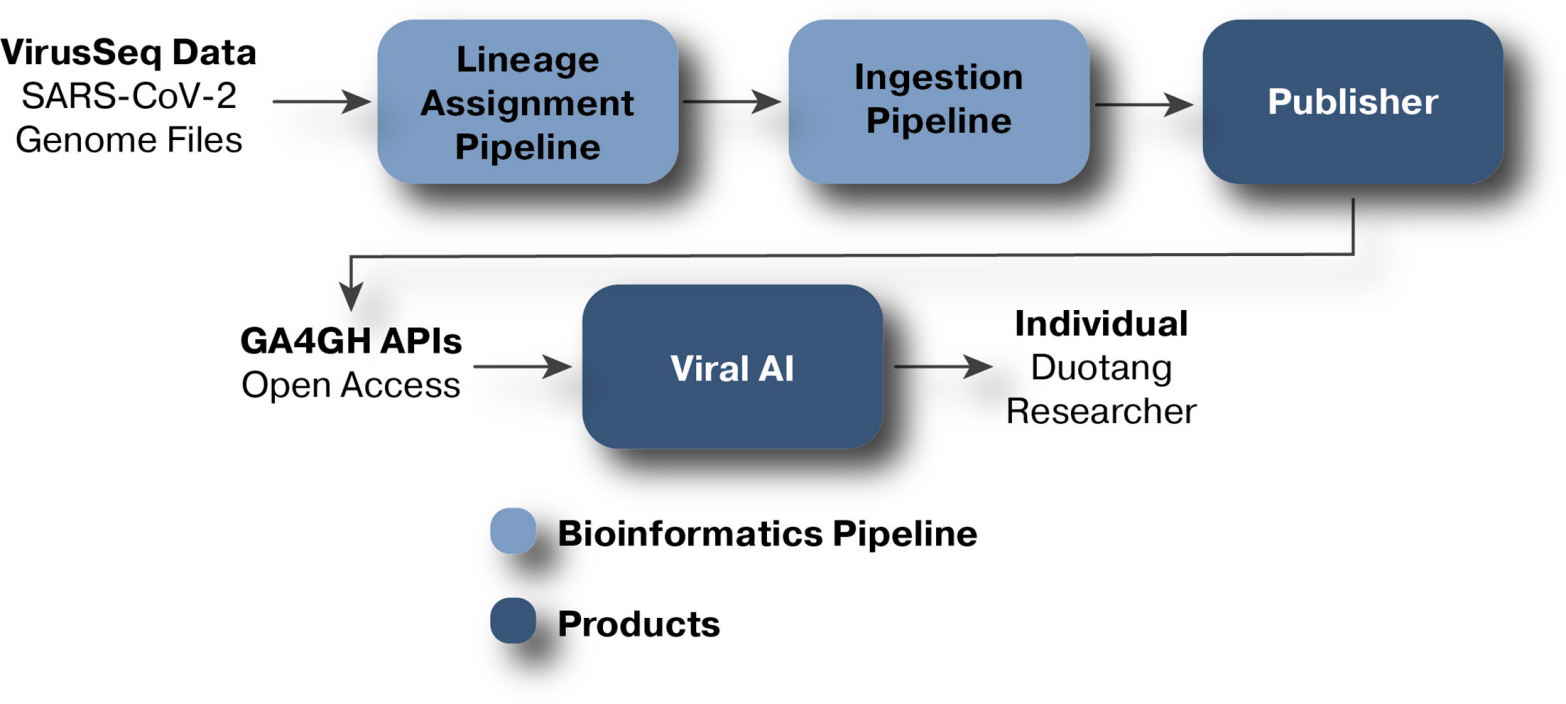
Overview of the data flow from VirusSeq Data Portal to Duotang. Genomics and epidemiological data from VirusSeq are first processed by DNAStack’s Viral AI workflow. The result of this is a dataset containing SARS-CoV-2 lineage information. Duotang then retrieves this lineage information using Viral AI’s Global Alliance for Genomics and Health (GA4GH) compliant APIs for further analyses.

Viral AI, developed by DNAstack in response to the COVID-19 pandemic to support access to and facilitate discovery from SARS-CoV-2 data, was used for lineage assignments and additional data queries/tabular visualizations. Assembled SARS-CoV-2 genomes are periodically retrieved from the data portal and lineages are assigned using pangolin (Phylogenetic Assignment of Named Global Outbreak LINeages [17]) in UShER [18] mode to maximize accuracy of assignments. Additionally, since pangolin nomenclature and designations are continuously updated as new variants are sequenced and categorized, upon update to pangolin or its databases, all previously assigned lineages are reassigned using the latest information. In addition to assigning lineages, the pipeline also produces, for each assembly, the set of sites that differs from the SARS-CoV-2 reference genome. The resulting metadata, variant sites, assemblies, and multifasta are processed through an ingestion pipeline and connected to Viral AI, where the data are made publicly available for further analysis and interpretation. Following their ingestion into Viral AI, the lineage metadata are retrieved and added to the VirusSeq Data Portal (Fig. 2). Releases of the data are maintained so that historical data with older lineage calls may be retrieved: https://virusseq-dataportal.ca/releases.

### Data harmonization and upload to VirusSeq data portal

Data curation and harmonization are crucial steps for the ingestion of data into any database, including the VirusSeq Data Portal, to ensure that accurate and comprehensive contextual data (sample metadata, epidemiological and methods information) can be associated with SARS-CoV-2 lineages and variants. This process involves collecting, organizing, and managing genomic contextual data from different public health laboratories across Canada to ensure their accuracy, completeness, and reliability. This process requires careful attention to detail, as errors or inconsistencies in data can lead to incorrect conclusions and hinder scientific progress. Furthermore, data sharing permissions differ among public health jurisdictions, and data curators possess an understanding of the ethical, legal, and privacy concerns linked to various datasets and resources (such as databases with restricted access versus those accessible publicly). Within the VirusSeq Data Portal, a data curator collaborates with both the PHAC-NML and CPHLN to manage these aspects effectively. These curators have an understanding of the ethical, legal, and privacy concerns linked to various datasets and resources. The data curation and harmonization efforts in the VirusSeq Data Portal are supported by the DataHarmonizer tool [8] developed by The Centre for Infectious Disease Genomics and One Health (See ‘Data harmonization and upload to VirusSeq Data Portal’). The tool offers key features, such as contextual data harmonization, validation and quality assurance, offline functionality and local installation, support for collaboration, adaptability to different pathogens [e.g. antimicrobial-resistant (AMR) bacteria, mpox, influenza], and enhanced usability. This tool simplifies the process of uploading, organizing, and managing contextual data and FASTA sequences and performs validation to ensure that the genomic contextual data adhere to the metadata schema of VirusSeq Data Portal, preventing schema errors. These tools and templates have created a framework that enables analyses used to understand how the virus is transmitted through the population (e.g. Duotang and COVID-MVP [19], described below).

Additional validation and approval processes are able to identify situations where there are errors, such as duplicates, or the inadvertent inclusion of contextual data that could potentially lead to patient reidentification (such as precise geographical location of sample collection) when combined with other contextual data fields. The robust process of review and approval also empowers data submitters to have control and approval of their data before they are eventually made available to the public. Collectively, the portal combines robust quality checks, rigorous approval processes, additional contextual data, and fully open data, complementing other international resources.

### Duotang website for genomic epidemiology analyses and mathematical modelling

Duotang is a collaborative effort involving members of CoVaRR-Net’s CAMEO group. It is presented as a RMarkdown notebook summarizing ongoing investigations of the evolution and epidemiology of SARS-CoV-2 VOCs in Canada. On a weekly basis, genomic data and sample metadata are retrieved from the VirusSeq Data Portal and processed via the Duotang workflow. In addition, Canadian case count data are retrieved from the Canadian HealthInfo Database [20]. For phylogenetic reconstruction, the sequences are first aligned against the reference genome (GenBank accession NC_045512.2: SARS-CoV-2 isolate Wuhan-Hu-1) with minimap2 (v. 2.17; [21]) using a script adapted from CoVizu [22], and subsampled to (1) up to 10 000 samples for non-XBB lineages and (2) all samples from lineages that descend from the XBB recombinant, for which a separate tree is constructed. This sampling is performed three times. A maximum-likelihood tree is then reconstructed from each subsample using the COVID-19 release of IQ-TREE (v. 2.2.0; [23,24]), assuming a general time-reversible model (GTR; with unequal rates and unequal base frequencies) and parameters (-me 0.05 -nt 8 -ninit 10 -n 8). TreeTime [25] is used to reconstruct a time-scaled tree under a strict molecular clock model (the rate is estimated by TreeTime). The resulting trees are converted into an interactive web element within the RMarkdown HTML using ggfree (v. 0.2; [26]) and r2d3 (v. 0.2.6; [27]). Root-to-tip (RTT) regressions are performed by rooting the tree on the reference genome and then fitting robust linear models to the divergence and sample dates within each major clade. Interactive plots of RTT results and streamgraphs of variant frequencies are generated with r2d3 and custom JavaScript. For other plots (i.e. lineage frequency and selection), the metadata are ingested into R (v. 4.1.3) and transformed using dplyr (v. 1.1.4) and plotted using ggplot2 (v. 3.4.4). For interactivity of these plots within the notebook, Plotly R [28] is used. The RMarkdown notebook is compiled into an HTML page and hosted on Github Pages and CloudFlare Pages for the public. For private Duotang pages that can contain additional non-public data, the web page is password encrypted with Python (v. 3.9) and the Python library PyCryptoDome (v. 3.16) using SHA256 before being published via Github Pages.

### Estimating SARS-CoV-2 variant of interest (VOI) substitution rates

Substitution rates are obtained from the maximum-likelihood tree made using IQ-TREE (see ‘Duotang website for genomic epidemiology analyses and mathematical modelling’) and root-to-tip regression performed as described above (see ‘Duotang website for genomic epidemiology analyses and mathematical modelling’), without forcing the intercept to zero (similar results are seen when forcing the intercept). For the estimation of each VOI’s substitution rates over time, all sequences of that VOI present in the tree are used. While this ignores pseudo-replication among the samples due to relatedness, the estimated slope is robust, given the large sample sizes that capture multiple mutational events (see [29]). Standard error (se) bars are calculated based on the three independent sub-samples to reduce the influence of closely related viral samples. For comparison, a global rate estimate is obtained from a regression over time of all the sequences present in the tree, ignoring variant classifications.

### Estimating selection coefficients of variants of interest

Selection coefficients provide a measure of the rate of spread of a lineage, relative to other lineages. To estimate selection, we use standard likelihood techniques. In brief, sublineages of current interest are specified (e.g. XBB.1.5, EG.5.1, HV.1), and counts by day tracked over time. If selection were constant over time, the frequency of subtype *i* at time *t*, measured in days, would be expected to rise according to:

(1) $$p_{i}(t)=\frac{p_{i}(0)exp(s_{i}t)}{\Sigma_{j}p_{j}(0)exp(s_{i}t)}$$

where *s_i_* is the selection coefficient favouring subtype *i* per day and the index *j* ranges over the circulating subtypes.

To provide a consistent measure of selection, Duotang uses a specific variant as a reference over a window of time (e.g. 4 months), chosen to be the variant that predominated early in this window. The selection coefficient of this variant is set to 0, *s*_*i*_ = 0. Given a reference lineage to which a lineage is being compared, the same logic behind equation (1) gives:

(2) $$ln\left(\frac{p_{i}(t)}{p_{ref}(t)}\right)=ln\left(\frac{p_{i}(0)}{p_{ref}(0)}\right)+s_{i}t.$$

Thus, plotting the above over time (a logit plot), selection generates a linear rise over time, whose slope is the strength of selection *s_i_*. For example, a selection coefficient of *s_i_* = 0.1 implies that subtype *i* is expected to rise from *p*(0) = 10% to *p*(*t*) = 90% in frequency in 44 days (i.e. in 4.4/*s_i_* days), when considering only the subtype and reference. By contrast, other tools estimate selection on one lineage relative to all remaining lineages, as in equation (1) (e.g. the default setting in CoV-Spectrum [30]), but genetic changes in other lineages then cause selection to appear to vary over time.

**Fig. 3.**
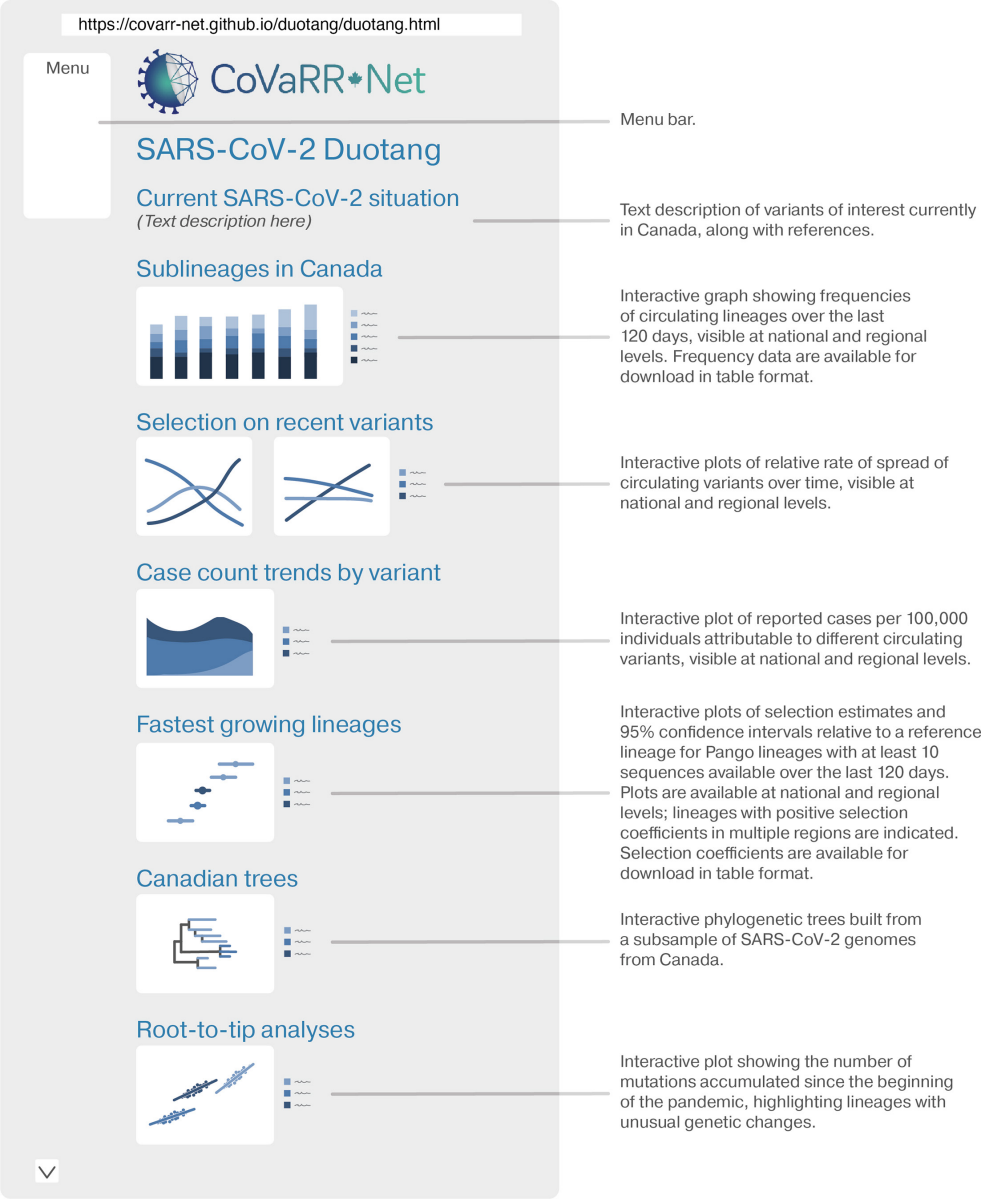
Duotang webpage overview. Duotang contains many different interactive plots for the user to explore SARS-CoV-2 genomic epidemiology in Canada. All sections of the page are easily accessible via the menu bar, which is located on the left-hand side of the page. The first section of the page gives a text description of current variants of interest in the country. Several of the plots are highlighted in the figure above, which include different visualizations of selection on variants, phylogenetic trees and root-to-tip analyses of these trees to detect unusual genetic changes. In addition to what is shown here, the user can also view plots of the growth advantage of single lineages relative to a reference lineage (on both national and regional levels), visualize the mutational composition of actively circulating lineages via an embedded frame of COVID-MVP [19], review the changing proportions of different variants over time (back to April 2020, on both national and regional levels), examine molecular clock estimates for different VOIs, and utilize a searchable table to view the ancestors and description of any Pango lineage. For more details, please visit https://covarr-net.github.io/duotang/duotang.html.

At any given time *t*, the probability of observing *n_i_*. sequences of sublineage *i* and *n*_ref_. sequences of the reference sublineage is binomially distributed, given the total number of sequences from that day and the frequency of each *p_i_*(*t*). Consequently, the likelihood of seeing the observed lineage frequencies over all times *t* and over both sublineages *j* is proportional to:

(3) $$L=\prod_{t}\prod_{j}{p_{j}(t)}^{n_{j}(t)}$$

assuming random sampling of cases and the evolutionary model [1]. The BBMLE (v. 1.0.25; [31]) package in R was used to maximize the likelihood of the observed data to estimate the unknown parameters *s_i_* and *p_i_*(0) relative to the reference (using the default optimization method, optim). For each selection coefficient, 95% confidence intervals are estimated by computing the inverse of the Hessian matrix obtained from the maximum-likelihood estimation. Then 95% confidence bands are obtained by randomly drawing 10 000 sets of parameters (*p_i_* and *s_i_* for each subtype) using RandomFromHessianOrMCMC [32], assuming a multinomial distribution around the maximum-likelihood point (estimated from the Hessian matrix). Graphs illustrating the rise in frequency of a variant over time are shown (see Fig. 3). Extensions comparing a set of variants to the reference is performed analogously, using a multinomial distribution and extending equation (3) for *j*>2.

To estimate case count trends by variant, reported cases obtained from Health Infobase Canada (weekly) or using provincial data sources (daily) where available (Alberta [33] and Quebec [34]) are normalized to cases per 100 000 individuals using Statistics Canada’s population estimates (27 September 2023 [35]). The last case data point for each region is then removed, as these data are still being gathered and are thus underestimated. The normalized cases over time (*n*^(*t*)^) are then log-transformed and fitted to a smooth spline with a lambda value of 0.001 using the smooth.spline() function within R.

The previously discussed methods allow us to estimate the proportion of a lineage over time [see equation 1 for *p_i_*(*t*) above]. Multiplying the frequency of a lineage by the total case count gives the inferred number of reported cases that are due to that lineage at each time point:

(4) $$n_{i}(t)=p_{i}(t)n(t)$$

Finally, once the inferred case counts (*n_i_*(*t*)) of each lineages are calculated, we take the last 2 days of data from the smooth spline times lineage frequency curve to obtain *n_i_*(*t*) and *n_i_*(*t*–1), from which the current exponential growth rate of that lineage (*r_i_*) is estimated as:

(5) $$r_{i}=ln\left(\frac{n_{i}(t)}{n_{i}(t-1)}\right)$$

The resulting estimates are then plotted using ggplot2.

## Results

### The VirusSeq Data Portal: an open-access SARS-CoV-2 sequence repository

**Fig. 4.**
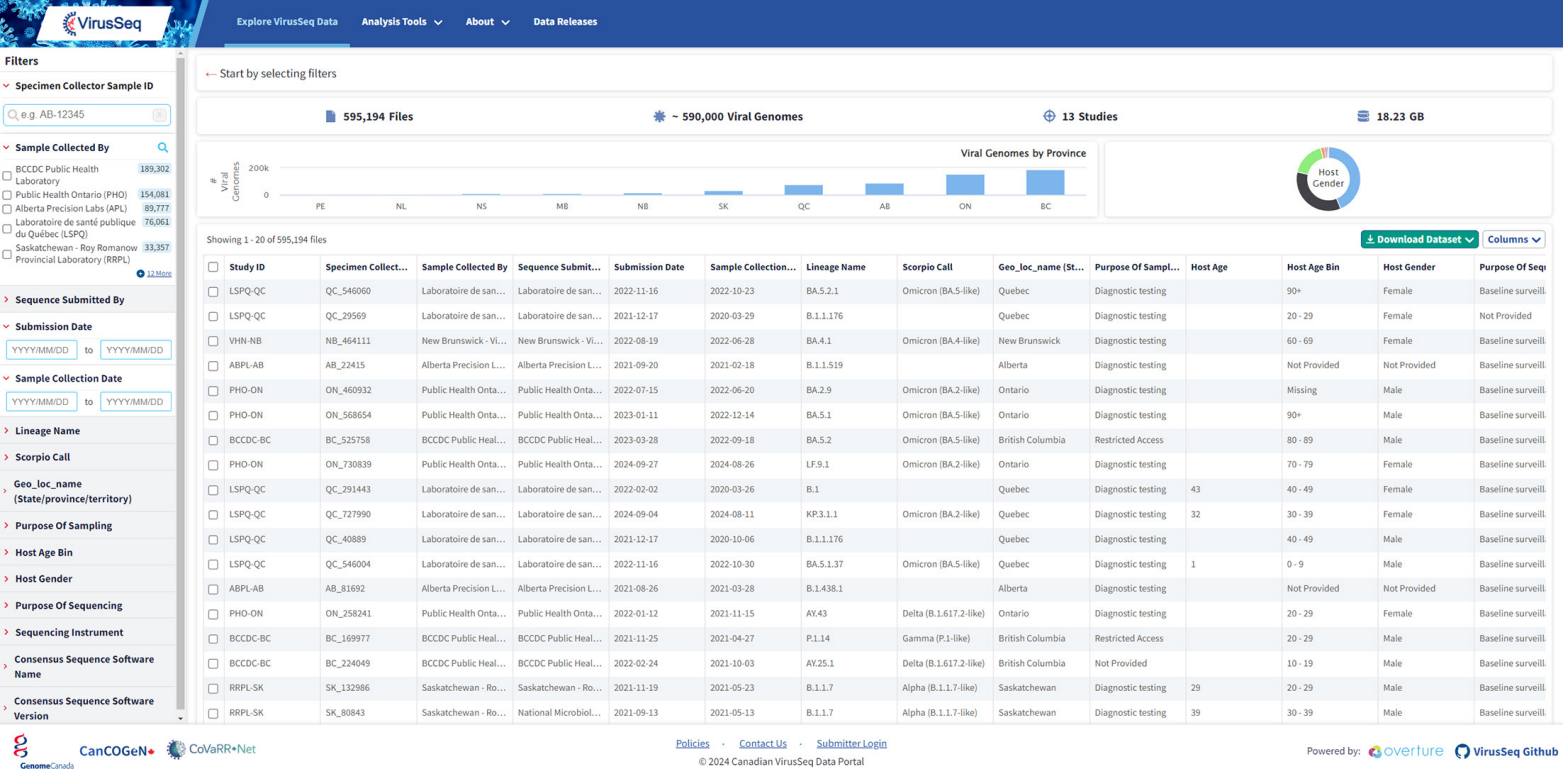
Overview of the VirusSeq Data Portal Explore page. The VirusSeq Data Portal allows users to browse samples stored via a web interface or API. Within the interface, the user is able to see the samples that are available and their metadata, and perform filters and queries to identify samples of interest that can then be downloaded for analysis.

In response to the COVID-19 pandemic, the initial version of the VirusSeq Data Portal database schema (https://virusseq-dataportal.ca/) was developed and deployed in a rapid 4 week timeframe (April 2021) (see ‘Development of the VirusSeq Data Portal’). The Ontario Institute for Cancer Research’s (OICR) repurposable open-source data management software that serves as a document management system–user interface, Overture, played a crucial role in streamlining the development process for the project. The modular architecture was particularly beneficial, allowing the system to scale in response to unexpected data submission surges. There were initial delays in receipt of data, as important data sharing agreements and procedures were developed that supported federated public health–academic collaboration. An ethics review and surveys of the public were also completed, to help inform appropriate data sharing policies [36,38]. As of March 2024, the data portal houses >550 000 SARS-CoV-2 viral genomes and their associated contextual data (metadata) collected across Canadian provinces, with more being added on a weekly basis. Typical updates are in the range of hundreds to thousands of sequences, with specific numbers for each week available at https://virusseq-dataportal.ca/releases. Contextual data include anonymized information regarding the patient from which samples were collected (e.g. age bracket, gender), geographical and temporal information (e.g. province/territory of origin, sample collection date), epidemiological information (e.g. purpose of sampling, purpose of sequencing), sequencing and bioinformatics information (e.g. sequencing protocol, dehosting method), viral lineage information, and a range of additional data fields (see Supplementary Information section 2.1, available in the online version of this article, for details). The portal satisfies the needs of three primary user groups: data administrators, submitters, and public users. Public users can access the data via an API or the user-friendly web portal, which facilitates data filtering using a diverse range of metadata fields (Fig. 4). Users can then download the filtered metadata alone or alongside the associated FASTA files. Data providers with authorization to submit have a dedicated graphical user interface to upload genomic data and associated metadata. Lastly, data administrators can access back-end services that control data access, enable maintenance and data configuration, and manage user authorization and authentication. The modular design of the data portal has already been reused in additional projects aiming to track pathogen genomics (e.g. [39]).

Lineage assignments, in combination with sample metadata and assemblies, are also imported into Viral AI, where they are made available over GA4GH (Global Alliance for Genomics and Health) standard interfaces. In addition to assigning lineages, the pipeline also produces, for each assembly, a set of sites that differs from the SARS-CoV-2 reference genome (see Supplementary Methods section 1.2). The resulting metadata, variant sites, assemblies, and multiFASTA files are processed through an ingestion pipeline and connected to Viral AI, with the data made publicly available for further analysis and interpretation (https://viral.ai/collections/virusseq/overview). Finally, metadata are retrieved and added to the VirusSeq Data Portal.

### Duotang: a multifaceted resource for tracking variants

Duotang is a webpage displaying interactive analyses of genomic and epidemiological data from the data portal and regionally reported case counts. Duotang is enabled by the steady stream of open-access, harmonized data deposited into the VirusSeq Data Portal (see ‘The VirusSeq Data Portal: an open-access SARS-CoV-2 sequence repository’ above). This deposition is powered by data generators across Canada who are willing to openly share anonymous data. The only criterion for use is to acknowledge the contribution of VirusSeq and its partners in any publication (see acknowledgements and consortium and network author information in the Supplementary Material section 3).

The various analyses and tools that Duotang offers include a manually curated text summary of the current situation, reviewed by multiple CAMEO members, plots of major sublineages that have been circulating in the country within the last 120 days, selection estimates on groups of lineages, case count trends by variant, selection estimates and 95% confidence intervals for the fastest growing pangolin lineages (see ‘Estimating SARS-CoV-2 Variant of Interest (VOI) substitution rates’ and ‘Estimating selection coefficients of variants of interests’ above), focusing on variants with more than 10 sequences in Canada over the past 4 months, and highlighting those undergoing positive selection in multiple provinces. It also shows phylogenetic trees, root-to-tip analyses, and molecular clock estimates. Most of these visualizations are interactive, allowing the user to select distinct lineages/sublineages, Pango groups, or plot types. Furthermore, Duotang is updated weekly, as data are being released in the VirusSeq Data Portal. The implementation of these analyses and their visualizations are described in the Methods section. Here, selected features of Duotang are briefly described. Please refer to Fig. 3 for an illustrated introduction to Duotang.

#### Current SARS-CoV-2 situation and summarizing variants

For each update, a snapshot summary of the current SARS-CoV-2 landscape within Canada is prepared by reviewers from CAMEO who are experts in molecular biology, microbial genomic epidemiology and mathematical modelling. Then the most frequent 50 sublineages of SARS-CoV-2 present in Canada are plotted, categorized by week. The frequencies are expressed both as an absolute sequence case count and as a relative count.

#### Estimating selection on variants of interest and integrating case counts

Duotang also provides a visualization of the estimated rate of spread of lineages, specifically, visualizing if new or emerging lineage(s) have selective advantages, and by how much, relative to a previous predominant lineage over the last 4 months. Thus, we can determine if new or emerging lineage(s) have selective advantage(s), and by how much. Selection coefficient estimates have become standard metrics of new variant fitness and an early indicator of potential impact, which are now incorporated in global technical reports, such as those provided by the US Centers for Disease Control and Prevention [40] and CoV-Spectrum [41]. Tracking selection coefficients can show accelerating or decelerating spread. Such changes in selection have been observed following widespread reductions in transmission, such as following a ‘circuit breaker’ in British Columbia Canada on March 30 2021 aimed at halting the spread of Alpha and Gamma [41]. Outbreaks or other localized transmission can also lead to a rise in the frequency of a particular variant, but such a rise is restricted in time and place. Duotang, thus, highlights those variants that are observed to rise in frequency across time – and across multiple provinces – allowing variants with a probable true selective advantage to be distinguished from variants that rise in frequency by chance. For example, in late autumn 2023, it became clear that JN.1 was showing a notable growth advantage in multiple provinces (see Fig. S2). Moreover, by integrating this selection coefficient with clinical PCR-based case data, or any measure of case data, we can estimate the proportion of cases that can be attributed to a specific variant and the current growth rate of each variant (Fig. 3).

#### Phylogenetic trees, root-to-tip analyses, and molecular clock estimates

Duotang also presents interactive phylogenetic-based analyses, including time and diversity trees and molecular clock estimates (Fig. 3). The interactivity of these analyses allows the user to quickly identify samples that are outliers but may hold clinical significance, (e.g. identification of highly divergent sequences or lineages that have re-emerged after a long absence, both of which may indicate passage through a chronic infection or another host species) [42,44]. Furthermore, the slope of the root-to-tip plots over time provides an estimate of the substitution rate. A lineage with a steeper positive slope than average for SARS-CoV-2 is accumulating mutations at a faster pace, while a lineage that exhibits a jump (a shift up in intercept but not slope) has accumulated more than expected numbers of mutations given the time since divergence from its parent lineage (similar to Alpha when it first appeared in the UK) [29,41]. We can use this plot to also identify sudden reappearances of previously predominant variants. For example, by selecting in the root-to-tip plot only the Delta variants, one can observe the occurrence of a sample classified as a Delta variant in July 2023 (a Delta wave predominated in autumn 2022 in Canada, so this was not expected), which upon further investigation was from a chronically infected individual (see Fig. S3).

### Additional resources

In addition to Duotang, the open-source data within the VirusSeq Data Portal have also powered other tools. One such example is CoVizu, which illustrates the mutational connections among sampled lineages (https://virusseq-dataportal.ca/visualization; https://github.com/PoonLab/CoVizu) [22]. Another is COVID-MVP (https://virusmvp.org/covid-mvp/) [19], which connects mutations with their functional impact. It is an interactive heatmap-based visualization app that allows users to explore the mutational profile of a set of genomes, including the underlying literature on the functional impact linked with each mutation. Functional annotation is a continuous effort that is primarily performed by curators working on the CAMEO initiative, but the scientific community can also provide input by using the issue tracking system of the GitHub repository (https://github.com/nodrogluap/pokay), where the annotations are maintained. Comprehensive details on COVID-MVP features, including the visualization, backend genomics workflow, functional annotations, and deployment of the framework to individual systems or cloud-based HPCs, are described in [19].

### Ethical considerations for the VirusSeq Data Portal

Article 27 of the 1948 Universal Declaration of Human Rights guarantees the rights of every individual in the world “to share in scientific advancement and its benefits” [45]. The sharing of genomic and health-related data is of key importance for pandemic monitoring and prevention for the benefit of human health. The VirusSeq Ethics and Governance Working Group drafted a policy memorandum along with a scientific manuscript [36] to clarify the Canadian ethical and legal framework applicable to the privacy implications of sharing viral genetic sequences and engage the Canadian public health and research community and other stakeholders. The conclusion of this work was that the sharing of viral genetic sequences that have been carefully dehosted of any human-like or non-viral sequences through the application of robust technical standards is unlikely to create any privacy risk for the host of the virus. Adding to this information a minimal amount of non-identifying metadata (e.g. age binned by category, gender, instrument used for the collection, etc.) does not change this general conclusion. By providing clear and concise information on the legal and ethical aspects of data sharing, the working group aimed to have a positive impact on Canadian practice.

To ensure the VirusSeq Data Portal would be compliant with the highest ethical and legal standards, we created a data security committee responsible for assessing the security, integrity, privacy, and ethical compliance of the portal in an ongoing and forward-looking manner. The committee participated in all team meetings, allowing any ethical or legal concern to be quickly addressed by the portal team. The presence of a data security committee aimed to increase the confidence of stakeholders contributing data to the portal.

## Discussion

### Benefits of open data

The VirusSeq Data Portal and Duotang are open-source and open-access resources that allow users to discover and download SARS-CoV-2 genomic data and explore mathematical models and genomic epidemiology analyses performed on the aforementioned data. The VirusSeq Data Portal simplifies Canadian SARS-CoV-2 genomic data discoverability and retrieval for users by providing a single, central repository where sequences are available from a federated, provincial health care system. Duotang allows users to see diverse types of analyses on an interactive webpage, preventing individuals from having to switch between various sites to find what they are looking for. While developed for Canada, the tools may be readily applied to other jurisdictions. The benefits of such open data have been described elsewhere [41] and can be summarized as (1) increasing scientific collaboration and innovation, (2) supporting research and potential for increased analyses, (3) informing public policy and decision-making, (4) allowing the public to be better informed, and (5) enabling scientific reproducibility. Indeed, the portal has permitted analyses [12,46] that would not otherwise have been possible. The portal has also allowed the development of Duotang, which is used as a resource by PHAC-NML, the CPHLN, and researchers who present modelling updates to government officials, thus informing the development of public policy and accelerating innovation. Industry has also used it to help inform vaccine development, and academics have used it to inform research priorities. None of this would have been possible without a commitment to open data by a range of Public Health and hospital workers and academic researchers. By raising the profiles of the data portal and Duotang via this paper, we aim to inform the broader research community of the existence of these resources, to promote, open, FAIR data, and to enable further analyses that have relied on the portal. See, for example, [12,19, 22, 46,49].

Multiple different jurisdictions around the globe have encountered their own COVID-19 data sharing challenges, and it is clear from the variety of repositories that have been developed (the VirusSeq Data Portal, the European COVID-19 Data Portal, California’s COVIDNet, NCBI’s SARS-CoV-2 Data Hub, and many others), that there is no ‘one size fits all’ approach. Each system has its own strengths and lessons learned that will shed light on resources developed/adapted for the next pandemic.

Duotang takes advantage of the open data available in the data portal to present the user with value-added analyses of genomic and epidemiological data that are easy to understand. While other tools provide some overlapping functionality [e.g. CoV-Spectrum [30] and the European Respiratory Virus Surveillance Summary (ERVISS) dashboard [50]], Duotang focuses on presenting data in a manner that does not require expert knowledge to interpret. CoV-Spectrum [30] is an excellent tool to estimate selection on SARS-CoV-2 lineages, but it is challenging to interpret because of the shifting suite of variants as evolution proceeds. By default, this tool measures selection on a lineage relative to all other lineages, which causes selection to vary over time because the background lineages continue to evolve (e.g. the selection coefficient for JN.1 appears to rise and fall over time). This spurious behaviour can be fixed by knowledgeable users by setting ‘Compare variants to a baseline’ (e.g. estimating selection of JN.1 compared to HV.1), but this requires expert knowledge on the part of the user. Specifically, the user needs to know the difference between selection measured relative to a group of variants versus one variant, they need to know the difference between measuring selection for lineages versus mixed clades (e.g. JN.1 versus JN.1*), and they need to know a relevant baseline variant, which needs to be appropriately chosen (co-circulating in time and well sampled). Duotang is semi-automated with expert input on these issues on a weekly basis (e.g. the baseline variant is updated as needed to ensure the most relevant variant serves as the comparator). In addition, Duotang provides selection coefficients for all the variants currently spreading in one convenient figure (available in table format as well), providing a quick overview of which variants are likely to take over in the near future. While ERVISS [50] does track ongoing evolution of SARS-CoV-2, it does not differentiate between circulating sublineages (e.g. all sublineages derived from BA.2.86 are grouped as BA.2.86).

### Challenges and solutions

In Canada, as in other countries, data silos existed at the beginning of the pandemic. This was largely due to the aforementioned federated nature of the Canadian provincial healthcare system. Once sequencing capacity was built and different regions began to produce their own data, regions faced uncertainty regarding the legal and ethical requirements applicable to data sharing and how to prevent the reidentification of patients, as well as about the kind of rigorous quality control steps that would ensure that only high-quality data were shared. These challenges were overcome through careful consultation and agreement on clear policies.

The existing contextual data that are available from the portal are sufficient to perform many useful analyses (such as those in Duotang), but additional (and linked) contextual data could allow us to determine (for example) how effective our vaccines are against current variants in a Canadian context, which variants spread more easily in certain contexts (such as among children), or which variants are more severe. Linking sequence data with epidemiological, clinical and immunization data would allow us to better utilize and leverage the sequence and minimal contextual data we already have [51]. This is not a simple undertaking in Canada, as these types of data are often collected by different parts of the health care system. For privacy reasons, linked data would have to be made available in a way that would afford reasonable privacy protection from reidentifiability to patients and research participants. A first study of Canadian participants' preferences on the matter indicates a general willingness to share their data in a pandemic context [37]. Another option would be to ensure that any linked, potentially identifiable data would be made available solely to vetted researchers.

### The future of Duotang and the data portal

Collectively, the portal and associated resources aim to provide open SARS-CoV-2 sequence data and analyses that have undergone rigorous quality checks, with harmonized contextual data from across the country that are more substantial than those available from other online databases. With the focus shifting back to other viruses of concern, Duotang is being adapted into a framework that is capable of performing similar analyses for other emerging pathogens. Currently, an extension of Duotang, which houses data for influenza virus and mpox virus, is being reviewed internally. Furthermore, with the emergence of pathogen surveillance via environmental methods (e.g. wastewater sampling), Duotang can be further extended with metagenomics data in mind. Similarly, the data portal can be retooled in the future to enable storage and comparison of wastewater data. These enhancements will enable Canadians and others internationally to access and analyse a wide variety of emerging pathogen surveillance data, develop tools to improve analyses using these data, and provide templates for additional bona fide services for tracking infectious diseases of concern.

## supplementary material

10.1099/mgen.0.001293 Uncited Supplementary Material 1.

## Abbreviations

AMR: anti-microbial resistance
API: application programming interface
CAMEO: computational analysis, modelling and evolutionary outcomes initiative of CoVaRR-net
CanCOGeN: Canadian COVID Genomics Network
CoVaRR-Net: Coronavirus Variants Rapid Response Network
CPHLN: Canadian Public Health Laboratory Network
ERVISS: European Respiratory Virus Surveillance Summary
FAIR: findable, accessible, interoperable, reusable
GA4GH: Global Alliance for Genomics and Health
GTR: general time-reversible
HPC: high performance computing
OICR: Ontario Institute for Cancer Research
pangolin: phylogenetic assignment of named global outbreak lineages
PHAC-NML: Public Health Agency of Canada's National Microbiology Laboratory
PHA4GE: public health alliance for genomic epidemiology
QA/QC: quality assurance/quality control
RTT: root-to-tip
SSOT: single source of truth
VOCs: variants of concern
VOIs: variants of interest
